# Impact of treatment history on drug resistance of metastatic colorectal cancer organoids

**DOI:** 10.1016/j.isci.2025.113801

**Published:** 2025-10-17

**Authors:** Maarten A. Huismans, Lidwien P. Smabers, Sascha R. Brunner, Arne van Hoeck, Demi van de Kaa, Ingrid A. Franken, Emerens Wensink, Jan Koster, Richard Volckmann, Onno Kranenburg, Miriam Koopman, Hugo J.G. Snippert, Jeanine M.L. Roodhart

**Affiliations:** 1 Department of Medical Oncology, University Medical Center Utrecht, Utrecht University, Utrecht, the Netherlands; 2 Center for Molecular Medicine, University Medical Center Utrecht, Utrecht University, Utrecht, the Netherlands; 3 Oncode Institute, Utrecht, the Netherlands; 4 Laboratory of Translational Oncology, University Medical Center Utrecht, Utrecht University, Utrecht, the Netherlands; 5 Laboratory of Experimental Oncology and Radiobiology, Cancer Center Amsterdam, Amsterdam University Medical Center, University of Amsterdam, Amsterdam, the Netherlands

**Keywords:** Health sciences, Medicine, Medical specialty, Internal medicine, Oncology, Biological sciences, Cancer

## Abstract

The treatment of metastatic colorectal cancer (mCRC) is impeded by drug resistance. We investigated how prior chemotherapy affects tumor genotype, phenotype, and resistance mechanisms by comparing 35 patient-derived metastatic organoids (PDOs) from pretreated and chemonaive patients with mCRC. Combining PDO drug sensitivity assessments with RNA and whole genome sequencing, we found PDOs from pretreated patients exhibited higher mutational load and more structural variants. Chemotherapy-related mutational signatures correlated with previous exposure. PDOs from oxaliplatin-resistant patients maintained this resistance, showing the upregulation of *ZNF300*, *TGM2*, and Hedgehog pathway enrichment. Acquired resistance to 5-FU and irinotecan was only partially captured, with irinotecan resistance linked to specific mutational signatures and deep deletions in common fragile sites, associated with distinct gene expression profiles. Our findings reveal that PDOs capture chemotherapy-induced genomic and phenotypic changes differently depending on the drug, suggesting varied mechanisms of acquired resistance involving both tumor cell-intrinsic properties and dynamic tumor cell states.

## Highlights

• PDOs from pretreated patients show increased mutations and distinct chemo-related signatures
• Oxaliplatin resistance is maintained in PDOs through tumor cell-intrinsic properties
• Deep deletions at CFS are linked to irinotecan resistance in PDOs from pretreated patients
• Gene expression patterns reveal distinct mechanisms for acquired vs. primary chemo-resistance

## Graphical abstract

**Figure:**
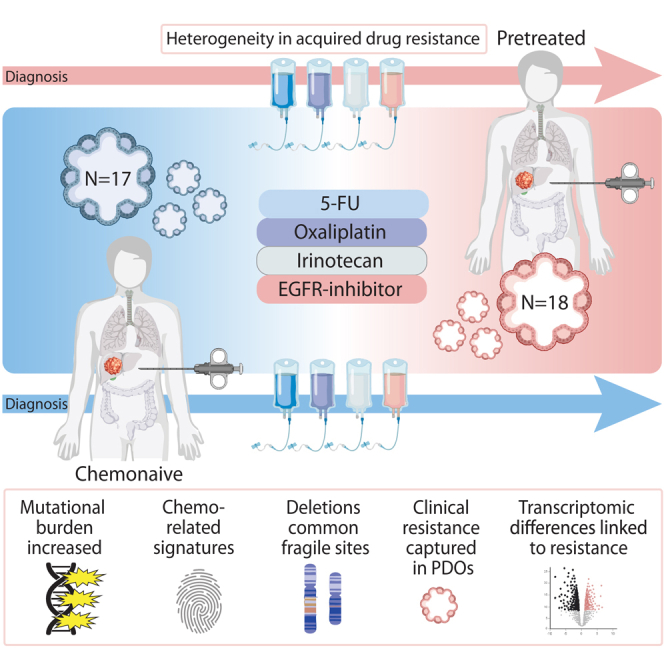

## Introduction

An important challenge in colorectal cancer (CRC) care is optimizing the systemic treatment of metastatic disease (mCRC). Specifically, to improve treatment efficacy, we need to increase the understanding of treatment resistance. Resistance can manifest as either primary resistance, where the tumor is inherently unresponsive, or acquired resistance, which develops as cancer adapts to treatment over time.^1^ Both tumor cell-intrinsic factors and the tumor microenvironment contribute to acquired resistance,^2^ and resistance can emerge through different pathways in different patients, making it complex to study. Factors influencing treatment resistance in patients include tumor burden, physical barriers, the immune system, and therapeutic pressure.^2^^,^^3^ To elucidate tumor cell-intrinsic resistance mechanisms, we need experimental models that phenocopy individual drug sensitivity while isolated from their micro-environment.

In recent years, patient-derived organoids (PDOs) have been increasingly adopted as experimental models that capture individual disease phenotypes.^4^ Foremost, organoids are scalable model systems that maintain histological, genetic, and transcriptional features of the original tumors.^5^^,^^6^ Additionally, the high success rate of establishing PDOs from individual tumors makes them promising avatar models to test drug responses,^7^^,^^8^^,^^9^^,^^10^^,^^11^ offering an experimental platform for personalized medicine.

At present, the effects of treatment history on tumor cell properties and resistance mechanisms remain poorly understood. Most studies linking patient response to *in vitro* PDO drug response have focused on chemonaive patients.^8^^,^^10^^,^^11^^,^^12^ In contrast, studies examining PDO drug response in pretreated patients are sparse.^13^^,^^14^^,^^15^ Understanding the tumor biology of heavily pretreated patients is crucial for advancing treatment strategies, particularly as these patients often participate in early-phase clinical trials evaluating novel therapies. Clinical studies on targeted therapy resistance in CRC show potential differences in resistance mechanisms between patients pretreated with chemotherapy and chemonaive patients: shifting from nongenomic or adaptive mechanisms to those driven by accumulated mutations.^16^^,^^17^^,^^18^ Single-cell RNA sequencing further demonstrates that tumors undergo changes during chemotherapy, as chemonaive tumors shift from conventional intestinal stem cell signaling to alternative pathways after first-line chemotherapy.^19^ Metastases from these patients exhibit increased plasticity and non-canonical transcriptional programs, linked to poor outcomes. It is unclear how a longer treatment history influences expression, whether acquired resistance mechanisms are unique to specific treatments, or whether there are shared adaptive pathways, and if these mechanisms can be modeled in PDOs.

We hypothesized that treatment history in patients leaves distinct footprints of resistance that can be modeled with PDOs. We studied 35 PDOs from patients with CRC, obtained either before the start of treatment or after exposure to standard-of-care treatment regimens. By integrating PDO drug sensitivity assessments with RNA sequencing and whole-genome sequencing (WGS), we directly compared molecular and functional properties between PDOs from chemonaive and pretreated patients. This approach revealed how treatment history influences tumor cell phenotype and drug resistance patterns, providing insights into both primary and acquired resistance mechanisms.

## Results

### Patient-derived metastatic organoids capture primary and acquired drug resistance

**Figure 1:**
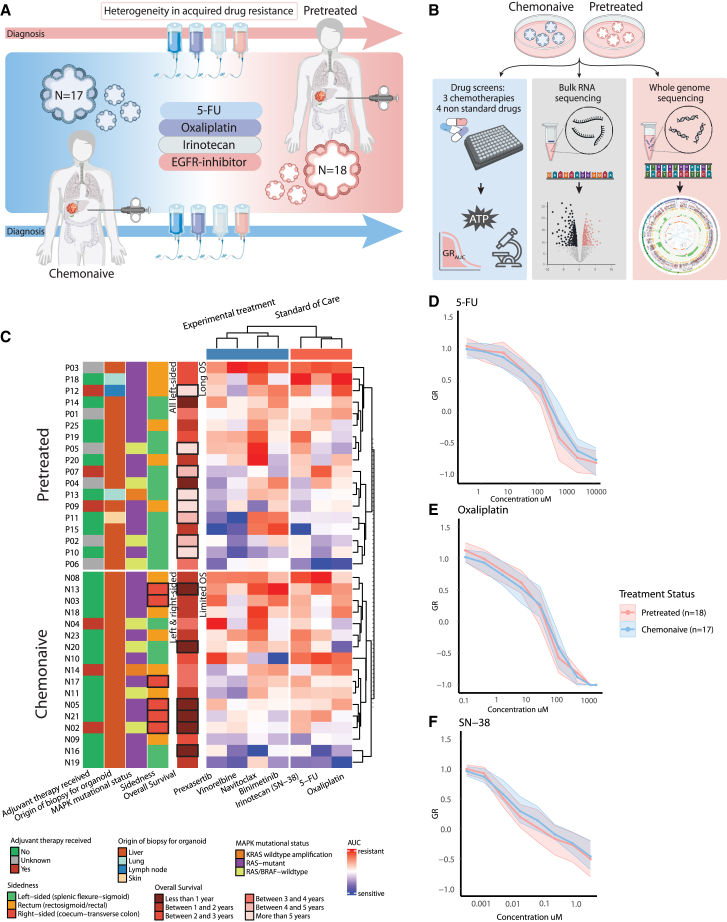
Overview of clinical and experimental data of PDOs derived from chemonaive versus pretreated patients with mCRC (A) Schematic diagram illustrates the timeline of the origin of the PDOs. Chemonaive PDOs were derived from CRC metastasis of patients immediately before starting their first line of palliative treatment. Pretreated PDOs were derived from CRC metastasis of patients after exposure to at least 5-FU, oxaliplatin, and irinotecan. (B) Graphic overview of different analyses to compare chemonaive and pretreated PDOs. (C) Heatmap of PDO drug response (normalized GR_AUC_) with patient and tumor characteristics shown on the left. *BRAF* non-V600E mutations are not shown in MAPK status. Rows represent individual PDOs (*n* = 35), stratified by treatment status (17 chemonaive versus 18 pretreated). Relevant differences in characteristics are annotated and enclosed with a box. For PDO response, the normalized GR_AUC_ is depicted for three standard-of-care treatments (SN-38, 5-FU, oxaliplatin) and four experimental treatments (prexasertib, vinorelbine, navitoclax, and binimetinib). (D–F) Mean drug response curves for standard-of-care therapies 5-FU (D), oxaliplatin (E), and irinotecan (SN-38, F) by treatment status: chemonaive (*n* = 17) and pretreated (*n* = 18). Y axis shows the growth rate (GR) metric, with 0–1 for partial growth inhibition, 0 for complete cytostasis, and 0 to −1 for cell death. Ribbons represent the interquartile range.

To investigate the development of chemotherapy resistance in mCRC, we used two groups of PDOs representing key stages in mCRC treatment (Tables S1 and S2). To compare mCRC PDOs from pretreated and chemonaive patients, we characterized the samples by assessing drug sensitivity (Figures 1A and S1A), gene expression patterns (bulk RNA sequencing), and genomic integrity (whole genome sequencing, Figure 1B). Most PDOs were derived from liver metastases (Figure 1C). For the chemonaive PDOs, biopsies were taken prior to first-line treatments. Most of these patients were found to be primary resistant to chemotherapy, as indicated by their limited median overall survival (17 months from first diagnosis of metastatic disease). Also, a high percentage of right-sided tumors was included in the chemonaive group (35%), which are known for their more aggressive and resistant phenotype. In contrast, the pretreated PDOs were obtained from patients who had a median overall survival of 42.5 months and included no right-sided CRCs. All pretreated patients were resistant to chemotherapy at the time of biopsy, but their relatively long time under treatment indicates an initial favorable response with acquired resistance over time. The clinical data of all patients is presented in Tables S1 and S2.

All PDOs were screened for sensitivity to standard-of-care (SOC) mCRC treatments, as well as experimental compounds prexasertib, vinorelbine, navitoclax, and binimetinib, that are tested in phase 1 trials for CRC (Figure 1C, Table S3).^20^^,^^21^^,^^22^^,^^23^^,^^24^^,^^25^ Overall, mean drug response (normalized GR_AUC_) for both PDO groups (chemonaive vs. pretreated) showed comparable sensitivity profiles to SOC chemotherapeutic agents (Figures 1D–1F) and experimental compounds (Figures S1B–S1F). This is in agreement with our assessment that both groups, albeit via different mechanisms, contain mainly PDOs from resistant CRCs. We found notable similarity in responses to CHK1 inhibitor prexasertib and SN-38 (Figure S1B, all treatments clustered). These drugs have been shown before to work synergetically in CRC cell lines.^26^

### Chemotherapy exposure correlates with deep deletions at common fragile sites, treatment-associated mutational signatures, and increased structural variants

**Figure 2:**
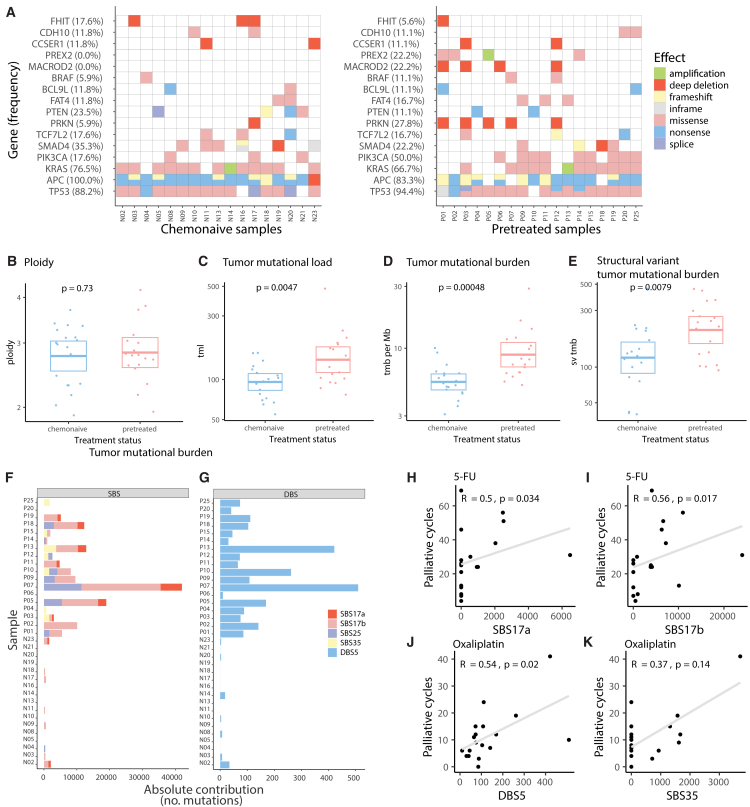
Differences in genomic alterations between PDOs derived from chemonaive versus pretreated patients with mCRC (A–E) Most prevalent somatic driver mutations are ordered from most to least prevalent in each group. All *BRAF* mutations are non-V600E mutations. B-E) Comparison of genomic characteristics between pretreated (*n* = 18) and chemonaive (*n* = 17) PDOs: tumor ploidy (B), tumor mutational load (tml, C), tumor mutational burden per megabase (tmb per Mb, D), and structural variant tumor mutational burden per megabase (sv tmb, E). (F and G) Contributions of single-base substitutions (SBSs) related to chemotherapies and G) oxaliplatin-associated double-base substitution 5 (DBS5). Oxaliplatin-associated DBS5 is present in all pretreated samples (*n* = 18) and minimally present (7/17) in chemonaive samples. Three of these seven chemonaive PDOs with DBS5 above average have been treated with adjuvant 5-FU and oxaliplatin. (H–K) Correlation of the number of 5-FU and oxaliplatin cycles patients received with the absolute contribution of respectively SB17a/b and SBS35/DBS5 in PDOs. Boxplots show the minimum, median, maximum, upper, and lower quartiles. Y axes of boxplots are on a logarithmic scale. Two-sided Student's t-tests were used to compare groups. Two-sided Spearman correlation tests were used for continuous variables.

To study the effect of prior treatment on the genome and transcriptome, we performed WGS and RNA sequencing on the PDOs from pretreated and chemonaive patients. First, we studied the presence of common CRC driver mutations in genes such as *TP53* and *APC* and confirmed that both groups are similar and representative of the stereotypic CRC in terms of their genomic landscape (Figure 2A). Next, we identified that deep deletions of driver genes located at common fragile sites (CFSs) tended to be more frequent in pretreated PDOs (44%, *n* = 8/18) compared to chemonaive PDOs (29%, *n* = 5/17). The most frequently affected driver genes at CFS were *MACROD2* (24% vs. 0%) and *PRKN* (29% vs. 6%). Deep deletions in other driver genes at CFS, including *NAALADL2* and *WWC3*, occurred in less than 5%.

We found no differences in overall ploidy between both groups and limited deviations between their karyotypes, most notably the more frequent loss of chromosome 8p in PDOs from chemonaive patients (Figures 2B and S1G). As expected, total mutational load and mutational burden were significantly higher in PDOs from pretreated patients (median 132 vs. 97 and 7.8 vs. 5.4, respectively, *p* < 0.01 for both, Figures 2C and 2D), as well as the number of structural variants (median 229 vs. 124, *p* < 0.01, Figure 2E).^27^ None of these factors were correlated with the number of treatment cycles (Figures S1H–S1J). We analyzed WGS data from a larger cohort of chemonaive (*n* = 78) and pretreated (*n* = 138) tumor samples, with similar treatment history.^28^ We confirmed a significantly higher mutational load and burden in pretreated patients (Figures S2A–S2D), validating the differences observed in PDOs.

To evaluate the genomic fidelity of PDOs, we compared six samples to their original tumors. The driver gene profiles showed 99% concordance (Figure S2E). Total mutational load and mutational burden were highly comparable across all samples (Figure S2F), confirming that PDOs closely resemble the tumor genome. As expected, tumor purity was around 100% in PDOs.

Mutational signature analysis provides insight into the patterns and etiology of DNA damage. Since chemotherapy agents cause distinct patterns of genomic alterations, we investigated treatment-associated single-base substitution (SBS) and double-base substitution (DBS) signatures to quantify prior drug exposure in patients from both groups.^29^^,^^30^^,^^31^^,^^32^ First, for all PDOs in our study, we classified their somatic mutations and assigned them to a mutational signature (Figure S2G). Then, we extracted the absolute contribution of SBS signatures that are linked to chemotherapeutic treatments: SBS17a and SBS17b to 5-FU,^29^ SBS25 to general chemotherapy exposure,^33^ and SBS35 to oxaliplatin (Figure 2F).^30^^,^^32^ As expected, we observed that these signatures were common in pretreated metastatic lesions (absolute contribution >1000 mutations in *n* = 16/18 samples) and rare in chemonaive (*n* = 2/17). Similarly, DBS5, which is linked to platinum,^34^ was mainly detected in pretreated PDOs (>10 absolute contribution in pretreated *n* = 17/18 vs. chemonaive *n* = 2/17, Figure 2G). We compared paired PDOs from the same patient before (N08), during (P26), and after treatment (P25). Comparison of these samples confirmed therapy-induced increases in total mutational load, mutational burden, and DBS5 (Figures S2I and S2J), while driver gene profiles remained comparable (Figure 2A).

The magnitude of treatment-associated mutational signatures in pretreated PDOs correlated with the number of chemotherapy cycles patients received. For instance, SBS17a and b contributions correlated to the number of 5-FU cycles received by the patient before PDO biopsy (R = 0.5, *p* = 0.03, and R = 0.6, *p* = 0.02, Figures 2H and 2I). DBS5 contributions correlated to the number of oxaliplatin cycles (R = 0.5, *p* = 0.02 Figures 2J and 2K). Intriguingly, not all patients who received 5-FU showed a 5-FU signature. In 13 out of 18 5-FU-pretreated PDOs (72%), SBS17b was identified, and in only 7 out of 18 (50%), SBS17a was identified. However, this apparent mismatch between treatment history and signature detection on the individual patient level is in line with previous characterizations of samples from 5-FU-treated patients.^27^^,^^29^^,^^35^^,^^36^ SBS35 contributions did not correlate significantly to oxaliplatin exposure (R = 0.37, *p* = 0.1).

In summary, WGS analysis revealed lasting genomic alterations in pretreated PDOs, including increased mutational burden, structural variants, and treatment-specific mutational signatures that correlated with prior drug exposure.

### Treatment-induced genomic alterations associate with increased SN-38 resistance

We used the treatment-induced genomic alterations as an indicator of effective drug exposure in patients. This approach allowed us to distinguish between tumor-intrinsic resistance and resistance due to external factors that limited drug exposure. When external factors prevent sufficient drug concentrations from reaching the tumor cells, this can mask potentially drug-sensitive tumors and result in an absence of drug-specific genomic alterations. We measured intrinsic drug sensitivity (GR_AUC_) profiles and compared them between pretreated PDOs with and without these alterations.

**Figure 3:**
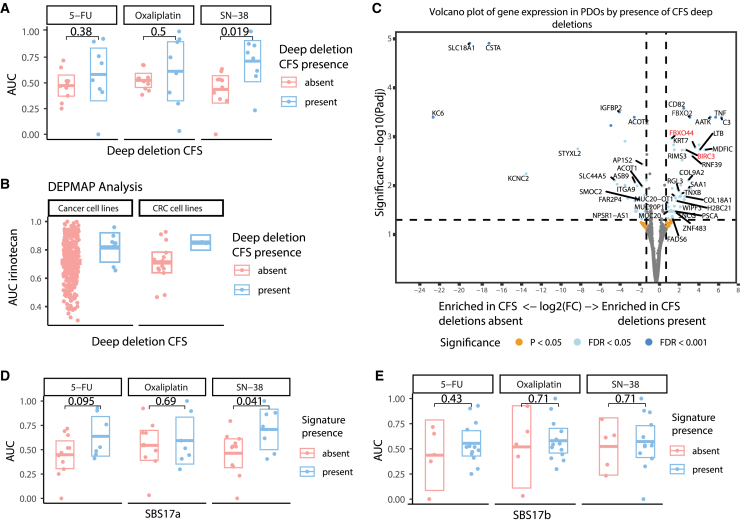
Treatment induced genomic changes influence drug resistance to SN-38 (A) PDO drug response (normalized GR_AUC_) to 5-FU, oxaliplatin, and SN-38 of PDOs by the presence of deep deletions in CFS (*n* = 8 with deletions, *n* = 10 without). (B) Drug response (cell viability) to irinotecan in DEPMAP cell lines by the presence of deep deletions in CFS: all cancer cell lines (*n* = 7 with deletions, *n* = 321 without) and CRC cell lines (*n* = 2 with deletions, *n* = 15 without). (C) Volcano plot of differential gene expression between PDOs by the presence of deep deletions in CFS. Genes in red are discussed in the text. (D) PDO drug response (normalized GR_AUC_) to 5-FU, oxaliplatin, and SN-38 by presence (*n* = 7) or absence (*n* = 11) of SBS17a. (E) PDO drug response by presence (*n* = 13) or absence (*n* = 5) of SBS17b. Boxplots show the minimum, median, maximum, upper and lower quartiles, and individual data points. Two-sided Student's t-tests were used to compare groups.

In patients, deletions in CFS have been associated with sensitivity to chemotherapy in general,^37^^,^^38^^,^^39^ and to SN-38 in particular, as it inhibits topoisomerase 1, which may play a role in CFS stability.^40^^,^^41^ However, within pretreated PDOs, those with deletions in CFS were significantly more resistant to SN-38 (median GR_AUC_ 0.77 vs. 0.49, *p* = 0.019, Figure 3A). No difference was observed with respect to oxaliplatin and 5-FU (*p* = 0.5 and 0.38, respectively). A similar trend of increased resistance to irinotecan in models with deletions in CFS compared to those without was observed using data from cell lines in the DEPMAP portal (Figure 3B).

When we compared transcriptional profiles for pretreated PDOs with and without CFS deletions, we found 105 significantly differentially expressed genes, 59 genes enriched in PDOs where CFS deletions were present, and 46 genes enriched in PDOs where CFS deletions were absent. Two notable genes enriched in PDOs with CFS deletions were *BIRC3* and *FBXO44*. *BIRC3* (8-fold change, FDR = 0.0019) is a gene associated with the inhibition of chemotherapy-induced apoptosis, which may explain the increased resistance to SN-38 *in vitro* (Figure 3C).^42^^,^^43^ Additionally, *FBXO44* (10-fold change, FDR = 0.00040*)* is a gene known to promote DNA replication-coupled repetitive element silencing and inversely correlate with replication stress in cancer cells, potentially representing an adaptive response that could contribute to SN-38 resistance.^44^

The 5-FU-related mutational signatures SBS17a and SBS17b were present in most, but not all, pretreated samples. We hypothesized that these signatures could indicate effective drug exposure during patient treatment. When comparing PDOs with and without these signatures, those with SBS17a showed a trend toward 5-FU resistance (*p* = 0.095) and were significantly more resistant to SN-38 (median GR_AUC_ 0.74 vs. 0.53, *p* = 0.04, Figure 3D). No differences in drug sensitivity were observed between PDOs with and without SBS17b (Figure 3E).

When we compared the gene expression of pretreated PDOs with and without SBS17a signatures, we found a limited number of differentially expressed genes, none of which were related to 5-FU exposure or topoisomerase sensitivity (Figure S2H). In conclusion, PDOs with deep deletions in CFS and chemotherapy-related mutational signatures were more resistant to SN-38, though it is unclear if these alterations drive resistance or just result from treatment exposure. While nearly all pre-treated patients in our study were resistant to SN-38, only a subset showed intrinsic resistance in PDOs. For these patients, the observed DNA and RNA changes may have contributed to their resistance mechanism.

### Oxaliplatin resistance persists in patient-derived metastatic organoids through non-genomic adaptations and the hedgehog pathway

**Figure 4:**
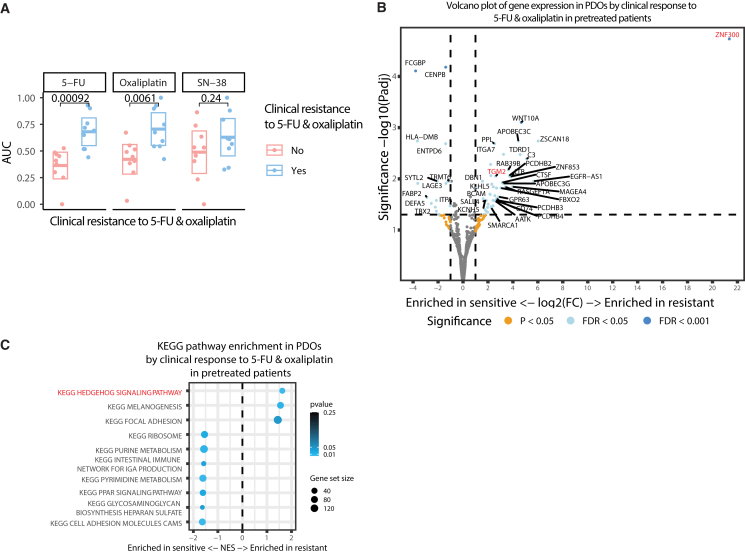
Clinically acquired resistance to 5-FU and oxaliplatin is captured in mCRC PDOs derived from pretreated patients (A) PDO drug response (normalized GR_AUC_) to 5-FU, oxaliplatin, and SN-38 of PDOs derived from patients who remained sensitive (*n* = 9) or developed clinical resistance (*n* = 9) to 5-FU and oxaliplatin. The absence of resistance development results in ongoing clinical response to oxaliplatin, categorized as sensitive. Boxplots show that PDOs from resistant patients are more resistant to 5-FU and oxaliplatin but not to SN-38. Boxplots show the minimum, median, maximum, upper, and lower quartiles and individual data points. Two-sided t-tests were used to compare groups. (B) Volcano plot of differential expression between oxaliplatin sensitive and resistant PDOs. Genes in red are discussed in the text. (C) KEGG pathway enrichment in PDOs by clinical response to oxaliplatin in pretreated patients. Pathways in red are discussed in the text. Dot size represents gene set size, and color intensity indicates statistical significance.

We showed that treatment-induced genomic changes in pretreated patients are linked to intrinsic resistance to SN-38. Next, we evaluated tumor cell-intrinsic resistance to oxaliplatin. We compared PDO sensitivity between PDOs derived from patients who were resistant to oxaliplatin (clinically refractory) versus PDOs from patients who had not developed clinical resistance to oxaliplatin (despite prior exposure). In line with their clinical manifestation, we found a significant difference in intrinsic PDO drug sensitivity for oxaliplatin (median GR_AUC_ 0.67 vs. 0.44, *p* < 0.01) and 5-FU (median GR_AUC_ 0.69 vs. 0.41, *p* < 0.01 Figure 4A). In contrast to SN-38, treatment-induced mutation signatures were not associated with cell-intrinsic resistance to oxaliplatin or 5-FU. Notably, unlike oxaliplatin, all pretreated patients were clinically refractory to 5-FU, and this resistance to 5-FU is not captured in all pretreated PDOs.

To gain deeper insights into acquired intrinsic resistance to oxaliplatin, we analyzed underlying differences in gene expression between PDOs derived from patients who remained sensitive to oxaliplatin versus those who developed resistance. The most dominantly enriched gene in PDOs from oxaliplatin-resistant tumors was *ZNF300* (log2 fold change 21, FDR <0.001, Figure 4B), which has previously been associated with platinum resistance in lung cancer cells.^45^^,^^46^^,^^47^ Another differentially expressed gene, *TGM2*, is also related to acquired CRC resistance and poor survival.^48^^,^^49^ KEGG pathway analysis revealed an enrichment of Hedgehog signaling in PDOs from oxaliplatin-refractory patients (Figures 4B and 4C). Gene expression profiles of longitudinally paired samples from one patient (before, during, and after treatment) were transcriptionally similar, indicating greater inter- than intrapatient variation (Figure S2K).

### Gene expression analysis identifies distinct signatures associated with primary versus acquired resistance

To explore whether differences in PDO drug sensitivity are related to differences in gene expression, we selected the 5 most sensitive and resistant PDOs from the complete cohort (chemonaive + pretreated) per treatment (Figure S3A). We identified 6 differentially expressed genes for 5-FU, 71 for SN-38, and 129 for oxaliplatin, with only 11 genes overlapping between SN-38 and oxaliplatin, and no overlap with 5-FU. *L1CAM* was one of the genes strongly differentially expressed between the 5 most sensitive and 5 most resistant PDOs to oxaliplatin (fold-change 4.8, FDR = 0.02) and SN-38 (fold-change 11, FDR = 0.01; corresponding log2FC values in Table S4). When comparing L1CAM expression with drug sensitivity (GR_AUC_), we found a consistent correlation with oxaliplatin resistance across all PDOs (R = 0.51, FDR = 0.045, Figure S3B).

When examining correlations between the expression of the differentially expressed genes (DEGs) and drug response across all PDOs, we found distinct patterns: 78 genes correlated with oxaliplatin resistance, while only 7 and 3 genes correlated with 5-FU and SN-38 resistance, respectively (Figure S3C, Table S5). Notably, the correlation with oxaliplatin held for all PDOs regardless of treatment history, whereas for 5-FU and SN-38, four and three genes, respectively, showed correlations exclusively in PDOs from pretreated patients. This pattern was further confirmed when analyzing pretreated and chemonaive groups separately, comparing DEGs identified from the 5 most sensitive versus 5 most resistant PDOs in each subgroup (Table S5). We found 82 unique genes from pretreated PDOs (accounting for 91 significant correlations with the three drugs) showing strong correlations with drug sensitivity of 5-FU and SN-38 in pretreated PDOs only (Figure S3D, Table S5). Furthermore, only 2 DEGs from chemonaive PDOs were associated with drugscreen outcomes (Figure S3E, Table S5).

To validate the clinical relevance of our findings, we examined gene expression in primary tumors from stage 3 and 4 CRC patients who received chemotherapy using a publicly available dataset.^50^ Survival analyses revealed that seven genes identified in the correlations between gene expression and PDO sensitivity or resistance showed significant associations with patient outcomes (FDR <0.1, Figures S3F–S3L). High expression of *ETS1*, *L1CAM*, *MIR31HG*, *NRIP3*, and *NDEL1* was significantly associated with worse overall survival. Conversely, *PPP1R1B* (Figure S3L) expression was associated with improved survival outcomes. Lastly, immunohistochemistry analyses supported our transcriptomic findings by showing that L1CAM expression, one of the genes associated with oxaliplatin resistance, was upregulated at both the RNA level in PDOs and protein levels in matched tumor tissues (Figure S4).

### Weighted gene co-expression network analysis identifies co-expression modules correlating with pretreatment status and drug response profiles

To further explore complex patterns of gene expression associated with drug resistance, we performed weighted gene co-expression network analysis (WGCNA).

First, we filtered gene expression data to retain the most informative genes (top 40% by variance, Figure S5A), providing sufficient information for meaningful analysis while excluding genes with little variance. Network stability (scale independence >0.8) and gene connectivity (Figures S5B and S5C) guided our selection of a soft thresholding power of 14. This filtering approach yielded 7905 genes. Sample clustering based on Euclidean distance between gene expression profiles (Figure S5D) confirmed the absence of spurious outliers.

**Figure 5:**
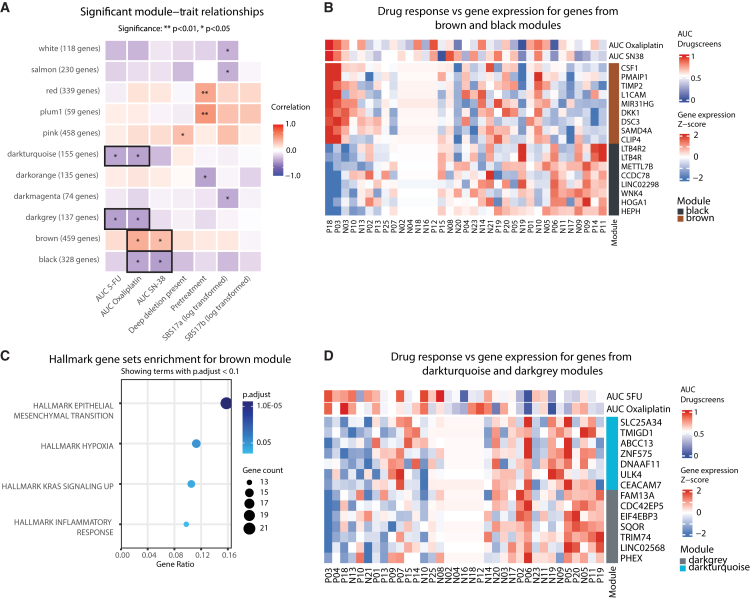
WGCNA analysis reveals gene modules associated with drug resistance in mCRC PDOs (A) Heatmap shows correlations between gene modules (rows) and clinical characteristics (columns) across PDOs (*n* = 35). Positive correlations in red, negative in blue. Asterisks indicate nominal significance from Student’s t-tests for correlation (∗*p* < 0.05, ∗∗*p* < 0.01). (B) Heatmap shows the expression of key hub genes from brown and black modules across PDOs. Brown module genes positively correlated with oxaliplatin and SN-38 resistance, while black module genes correlated with sensitivity. Expression is shown as the *Z* score. (C) Hallmark gene set enrichment for brown module. Dot size represents gene count, and color intensity indicates statistical significance. (D) Heatmap shows the expression of key hub genes from darkturquoise and darkgrey modules across PDOs. Both modules correlate with 5-FU and oxaliplatin sensitivity.

We identified 38 initial modules, which were reduced to 31 distinct gene modules after merging similar clusters, with each assigned a unique color identifier. When correlating these modules with clinical characteristics and drug response metrics, we observed 15 significant module-trait relationships (Figure 5A).

Three modules were correlated with pretreatment status (red, plum1, and darkorange). The plum1 module, positively correlated with pretreatment, contained key genes such as *CYP24A1*,^51^^,^^52^ *FOXQ,*^53^^,^^54^ and *TIMP3*.^55^^,^^56^ The darkorange module, negatively correlated with pretreatment, was enriched for cell-cell interactions, with key genes including *SCGN*, known to promote cell apoptosis and inhibit migration/invasion in CRC cells.^57^^,^^58^

The pink module was correlated with the presence of deep deletions in CFS and enriched for IFN-γ response and TNFR2 non-canonical NF-κB signaling. Furthermore, the module contained 20 genes overlapping with our DEG analysis in Figure 3B, including *FBXO44*, *BIRC3*, *TNF*, and *SAA1*.

Most importantly, four modules showed significant correlation with drug response (Figure 5A): brown and black correlated with oxaliplatin and SN-38 resistance and sensitivity, respectively (Figure 5B, Tables S6 and S7), and darkturquoise and darkgrey (Figure 5D, Tables S8 and S9) correlated with 5-FU and oxaliplatin sensitivity. The darkturquoise module was enriched for xenobiotic metabolism and drug metabolism via cytochrome P450, suggesting a role in drug detoxification (Tables S10 and S11). The dark grey module was enriched for IL6-JAK-STAT3 signaling and apoptosis pathways. The brown module was enriched for epithelial-mesenchymal transition, hypoxia, KRAS signaling, and inflammatory response pathways (Figure 5C, Tables S10 and S11), with key genes including *CSF1*, *TIMP2*, and *L1CAM*. The black module was enriched for peroxisome and *WNT*/β-catenin signaling pathways (Tables S10 and S11).

Our gene expression analysis reveals two key insights: 1) pretreatment in patients has a lasting effect on the transcriptomic landscape of derived organoids, validating PDOs as models that preserve treatment history; and 2) oxaliplatin resistance mechanisms are consistently “hardcoded” across all PDOs regardless of treatment status, while 5-FU and SN-38 resistance patterns are primarily observed in PDOs from pretreated patients, indicating distinct mechanisms of acquired versus intrinsic drug resistance.

## Discussion

Finding treatments for refractory CRC is challenging, with most phase 1 trials failing due to lack of efficacy.^59^ While drug development typically uses chemonaive tumor models, these lack potential tumor cell-intrinsic changes caused by prior treatments that may drive resistance. We analyzed PDOs from patients with extensive treatment histories and chemonaive PDOs to examine how treatment history influences the genomic landscape, gene expression, and tumor cell-intrinsic drug sensitivity. We found that chemotherapy induced the accumulation of genomic mutations and phenotypic changes that are related to exposure level and resistance. These changes are captured in PDOs. Oxaliplatin resistance is maintained in PDOs and mostly determined by static cell intrinsic changes. In contrast, resistance to irinotecan and 5-FU seems more related to transiently induced transcriptional changes under the influence of external factors.

We observed that chemotherapy exposure induces specific DNA alterations in most patients, though with significant inter-patient heterogeneity. These alterations are characterized by the accumulation of treatment-specific mutational signatures and deep deletions at common fragile sites. The relative abundance of these mutational signatures correlated with the duration of prior therapy and confirmed that the DNA-damaging agents truly reached the tumor.

For oxaliplatin, acquired resistance in patients is consistently captured in the pretreated PDOs, suggesting a predominant tumor cell-intrinsic resistance mechanism. We found that oxaliplatin resistance is not associated with specific genomic alterations, but it displays characteristic transcriptional changes involving the expression of *TGM2*,^48^^,^^49^^,^^60^ and enrichment of genes involved in focal adhesions,^61^ and Hedgehog signaling. Among these, *ZNF300* was particularly highly expressed in oxaliplatin resistant CRC PDOs. While *ZNF300* has been previously associated with therapy resistance in several cancer types, its potential role in oxaliplatin resistance in CRC merits further investigation.^45^^,^^46^^,^^47^^,^^62^ Previous studies have shown that high TGM2 expression correlates with poor progression-free survival and overall survival.^49^ Additionally, the inhibition of TGM2 in cell lines has demonstrated therapeutic potential, further supporting its role as a target for overcoming resistance in CRC,^60^ with compounds such as levamisole representing potential candidates for targeting its activity.

For SN-38, acquired resistance in pretreated patients was not captured in all PDOs, suggesting that external factors could mask tumor cell-intrinsic sensitive phenotypes in patients. PDOs from tumors with SN-38-related genomic alterations (deep deletion in common fragile sites and SBS17a) show increased resistance. This is likely due to permanent, DNA damage-induced mechanisms reflected in specific transcriptional changes involving the expression of *BIRC3* and *FBXO44*. Tumors from which PDOs lack these genomic alterations may have developed *in vivo* resistance through extrinsic or inducible mechanisms rather than permanent acquired traits. Notably, *BIRC3* represents a potential therapeutic target as an inhibitor of apoptosis protein, with IAP inhibitors such as Xevinapant currently in clinical development.

Acquired 5-FU resistance in pretreated patients could also not be captured in all pretreated PDOs. Further investigation is needed to determine if this reflects mechanisms that are not fully manifested in PDOs. This may involve interactions with the tumor microenvironment or transient, adaptive responses difficult to capture in *in vitro* models. For instance, cancer-associated fibroblasts are known to promote processes such as epithelial-mesenchymal transition, which is an important determinant of 5-FU resistance in CRC.^63^

Building on the identification of DEGs linked to resistance, we explored whether these genes correlate with drug resistance, and if these resistance phenotypes are distinct between primary and acquired resistant tumor cells. We found that primary and acquired resistance-related genes are distinct for 5-FU and SN-38, whereas they overlap for oxaliplatin. Pretreatment had a lasting impact on gene expression, reinforcing PDOs as models that preserve treatment history. We identified distinct gene expression modules associated with pretreatment status and drug resistance patterns in PDOs. Key resistance-associated modules were enriched for pathways linked to epithelial-mesenchymal transition, inflammation, drug metabolism, and apoptosis, highlighting potential therapeutic targets. The functional roles of many genes remain to be fully elucidated, warranting further detailed investigations in future studies.

Understanding distinct resistance mechanisms reveals targets to overcome chemotherapy resistance, forming a basis for developing strategies for therapy-resistant mCRC. PDOs capturing resistance traits highlight their potential as predictive models for chemotherapy-resistant patients. Incorporating such models into drug development could improve the identification of effective treatment strategies for patients with limited options.

### Conclusions

The chemotherapeutic treatment of patients with mCRC significantly alters the genome and transcriptome of tumors, and these changes are captured in PDOs, where they lead to resistance to treatment *in vitro*. Resistance mechanisms are treatment specific, with distinct genomic changes correlating with drug responses to different therapies. Acquired resistance to oxaliplatin in patients is maintained in PDOs through static tumor cell-intrinsic properties. In contrast, acquired resistance to irinotecan and 5-FU is only partially captured in PDOs with a greater role for dynamic, transient cell states or tumor cell extrinsic factors. This heterogeneity in resistance underscores the importance of incorporating tumor models that recapitulate drug-induced resistance in drug development for chemotherapy-resistant patients.

### Limitations of the study

Our study provides insights into both primary and acquired resistance mechanisms, yet it is limited by several factors. First, we focused on tumor cell-intrinsic mechanisms, but resistance can also be influenced by external factors such as interactions with the tumor microenvironment. PDOs are scalable and robust models that allow us to link tumor cell-intrinsic changes to drug sensitivity. However, they do not fully capture the influence of stromal or immune components, which can contribute to therapy resistance. While organoid co-cultures are becoming increasingly sophisticated,^64^^,^^65^^,^^66^ integrating and optimizing all TME components remains complex. Future research should explore advanced co-culture systems or animal models to better replicate the *in vivo* tumor environment and provide a more comprehensive understanding of resistance mechanisms. Second, extended cell culture to expand PDOs may gradually alter gene expression patterns, interfering with original tumor traits,^67^ and limiting our ability to identify transient resistance mechanisms. Third, while we identified correlations between transcriptomic changes and drug sensitivity, we cannot infer causality from these associations alone. Further functional studies are needed to confirm these links and their role in resistance development.

Several design considerations influenced our approach and findings. Ideally, establishing paired PDOs sampled before and after chemotherapy would allow direct study of the emergence of acquired resistance and enhance mechanistic understanding while eliminating interpatient variability in gene expression and drug sensitivity. However, this approach presents logistical challenges and is often unfeasible in clinical practice. Additionally, cultural success varies based on clinical characteristics, introducing potential selection bias. We prioritized a larger sample size over paired comparisons to ensure broader representability. Although PDOs established from single biopsies may not fully capture tumor heterogeneity, which may affect drug sensitivity,^68^ previous studies have demonstrated that intrapatient variability outweighs interpatient variability in drug sensitivity and gene expression patterns.^69^ Our transcriptomic data support this observation, with gene expression clustering primarily by patient, where multiple samples per patient were available. Finally, as all patients in our study were fit for biopsy and either early-stage or heavily pretreated, caution is warranted when extrapolating these findings more broadly.

In our cohort, sex distribution differed modestly between groups: the chemonaive arm included 59% male patients (10 of 17), and the pretreated arm included 74% male patients (14 of 19). These differences likely reflect the underlying incidence and referral patterns of patients participating in oncology trials,^70^ rather than sex-specific biology. We did not specifically investigate sex effects in our study, but consider it unlikely that the differences in sex distribution materially influenced our results, as our analyses focused on the individual effects of genomics and transcriptomics on treatment resistance in organoids. We acknowledge this as a limitation and encourage future studies with larger sample sizes to examine potential sex-related effects.

## Resource availability

### Lead contact

Requests for further information and resources should be directed to and will be fulfilled by the lead contact, Jeanine Roodhart (j.roodhart@umcutrecht.nl), University Medical Center Utrecht, Heidelberglaan 100, 3584 CX Utrecht, the Netherlands.

### Materials availability

This study primarily utilized PDOs from patients with mCRC. These PDOs were established following the protocol described by Van de Wetering et al.^5^ While there are no unique reagents created specifically for this study, researchers interested in accessing the PDO lines should contact the lead author with a completed materials transfer agreement due to the patient-derived nature of these materials.

### Data and code availability

• The RNA sequencing, whole genome sequencing (WGS), and drug screen datasets generated in this study are available in R2: Genomics Analysis and Visualization Platform (http://r2.amc.nl).^71^ via http://r2platform.com/strategic.
• Code used for analysis of the WGS data is publicly available via the Hartwig analytical processing pipeline (GitHub - hartwigmedical/pipeline5).
• All other data analysis was performed using standard R packages as detailed in the key resources table.

## Acknowledgments

We would like to thank HUB Organoids B.V. for their technical expertise and contributions in the generation and culture of the OPTIC organoids. We are grateful to Joris Hageman, Julian Buissant des Amories, and Ingrid Verlaan for their technical assistance in establishing and maintaining the RASTRIC organoid cultures. We thank Sander Mertens for his help in establishing the drug screening protocols. This work was financially supported by 10.13039/501100021821Oncode Institute (CPoC P2019-0026) and HUB Organoids B.V.

## Author contributions

MAH and LPS: conceptualization, data curation, formal analysis, investigation, methodology, software, visualization, writing original draft, and writing-reviewing and editing. SRB and AvH: formal analysis, methodology, software, and writing-reviewing and editing. DvdK: investigation and writing-reviewing and editing. IAF: methodology, visualization, and writing-reviewing and editing. EW: data curation and writing-reviewing and editing. JK and RV: data curation and software. OK and MK: writing-reviewing and editing. JMLR and HJGS: conceptualization, funding acquisition, supervision, and writing-reviewing and editing. All authors reviewed and approved the final article.

## Declaration of interests

The authors declare no known conflict of interest. The following interests may be considered as potential competing interests. J.M.L.R. grants/contracts: BMS, Pierre Fabre, Servier, Cleara Biotech, HUB organoids; Board Membership: Foundation Hubrecht Organoid Biobank. M.K. grants/contracts: Bayer, BMS, Merck-Serono, Pierre Fabre, Servier, Roche, Sanofi, Personal Genome Diagnostics. All grants were unrelated to the study and paid to individual institutions.

## STAR★Methods

### Key resources table

**Table:** 

REAGENT or RESOURCE	SOURCE	IDENTIFIER
**Antibodies**		

L1CAM/CD171 antibody	Invitrogen	MA1-46044

**Chemicals, peptides, and recombinant proteins**		

5-fluorouracil (5-FU)	Accord	N/A
A83-01	MedChemExpress	#HY-10432
Advanced (DMEM/F12) medium	Gibco	#12634-010
Alpelisib	Selleck Chemicals	S2814
B27	Gibco	#11530536
Basement Membrane Extract (BME)	Bio techne	#3533-010-02
Binimetinib	Selleck Chemicals	S7007
Dimethyl Sulfoxide (DMSO)	Thermo Fisher Scientific	#20688
Dispase	Life Technologies	#17105041
Dulbecco′s Phosphate Buffered Saline	Merck	D8537-500 ML
GlutaMAX	Gibco	#35050-038
HEPES Buffer	Santa Cruz Biotechnology	#sc-300789
Human recombinant EGF	Sigma-Aldrich	#E9644
Human recombinant FGFbasic	PeproTech	#100-18B
Human recombinant IGF-I	BioLegend	#590908
Lapatinib	Selleck Chemicals	S1028
N-acetylcysteine	Sigma-Aldrich	#A9165
Navitoclax	Selleck Chemicals	S1001
Noggin conditioned medium	In-house production	N/A
Oxaliplatin	Fresenius Kabi	N/A
Penicillin/Streptomycin	Gibco	#15070063
Prexasertib	Selleck Chemicals	S7178
R-spondin conditioned medium	In-house production	N/A
SN-38	Selleck Chemicals	S4908
TrypLE	Gibco	#12604021
Wnt surrogate	U-protein Express	#N001
Vinorelbine	Selleck Chemicals	S4269
Y-27632	MedChemExpress	#HY-10583

**Critical commercial assays**		

CC1 Antigen Retrieval Buffer	Roche	06414575001
CellTiter-Glo 3D	Promega	G9681
TaqMan OpenArray technology	Thermo Fisher Scientific	N/A
OptiView DAB IHC Detection Kit	Roche	06396500001
TruSeq RNA Stranded polyA kit	Illumina	N/A

**Deposited data**		

Drugscreen data	This paper	https://doi.org/10.34894/QWAR8P
WGS data	This paper	https://doi.org/10.34894/QWAR8P
RNA sequencing data	This paper	https://doi.org/10.34894/QWAR8P

**Experimental models: Cell lines**		

Pretreated metastatic colorectal cancer patient biopsies	RASTRIC trial	European registry no. 2019-004987-23
Chemonaive and pretreated metastatic colorectal cancer patient biopsies	OPTIC trial	Dutch registry no. NL61668.041.17

**Software and algorithms**		

BSgenome v1.74.0	Bioconductor	https://doi.org/10.18129/B9.bioc.BSgenome
BSgenome.Hsapiens.UCSC.hg38 v1.4.5	Bioconductor	https://doi.org/10.18129/B9.bioc.BSgenome.Hsapiens.UCSC.hg38
ComplexHeatmap v3.19	Bioconductor	https://doi.org/10.18129/B9.bioc.ComplexHeatmap
Cowplot v1.1.3	CRAN	https://doi.org/10.32614/CRAN.package.cowplot
DescTools v0.99.60	CRAN	https://doi.org/10.32614/CRAN.package.DescTools
DESeq2 v1.44.0	Bioconductor	https://doi.org/10.18129/B9.bioc.DESeq2
GenomicRanges v1.58.0	Bioconductor	https://doi.org/10.18129/B9.bioc.GenomicRanges
Ggalluvial v0.12.5	CRAN	https://doi.org/10.32614/CRAN.package.ggalluvial
Ggforce v0.4.2	CRAN	https://doi.org/10.32614/CRAN.package.ggforce
Ggplot2 v3.5.2	CRAN	https://doi.org/10.32614/CRAN.package.ggplot2
Ggrepel v0.9.6	CRAN	https://doi.org/10.32614/CRAN.package.ggrepel
Hartwig analytical pipeline	Hartwig Medical Foundation	GitHub - hartwigmedical/pipeline5
KM plotter	N/A	https://kmplot.com
Limma v3.60.0	Bioconductor	https://doi.org/10.1093/nar/gkv007
MutationalPatterns v3.16.0	Bioconductor	https://doi.org/10.18129/B9.bioc.MutationalPatterns
Nlme v3.1-168	CRAN	https://doi.org/10.32614/CRAN.package.nlme
Nplr v0.1-8	CRAN	https://doi.org/10.32614/CRAN.package.nplr
Pheatmap v1.0.12	CRAN	https://doi.org/10.32614/CRAN.package.pheatmap
R	R foundation	v4.0.3
VariantAnnotation v1.52.0	Bioconductor	https://doi.org/10.18129/B9.bioc.VariantAnnotation
WGCNA v1.71	CRAN	https://doi.org/10.32614/CRAN.package.WGCNA

**Other**		

Bioanalyzer	Agilent	N/A
D300E drug dispenser	Tecan	N/A
MultidropTM Combi Reagent dispenser	Thermo Fisher Scientific	N/A
NextSeq2000	Illumina	N/A
Novaseq6000	Illumina	N/A
QuantStudio 12K Flex Real-Time PCR system	Applied Biosystems	N/A
Ventana Ultra Benchmark	Roche	N/A
Spark Plate Reader	Tecan	N/A

### Experimental model and study participant details

#### Human participants

We selected mCRC PDOs from two groups of patients. The first group consists of patients that had a biopsy immediately before starting their first line of treatment for metastatic disease (hereafter referred to as “chemonaive”). The second group consists of patients that had a biopsy after clinical exposure to at least the following palliative treatment lines for mCRC: a fluoropyrimidine, oxaliplatin, irinotecan, and an EGFR and/or VEGFR inhibitor if indicated (hereafter referred to as “pretreated”). Biopsies were taken from liver, lung, lymph node or skin metastases, all microsatellite stable.

The pretreated group included 11 patients with a RAS-mutation who participated in the ongoing phase 1/2 RASTRIC trial (European registry no. 2019-004987-23) focusing on RAS-mutant mCRC, and 7 RAS-wildtype patients from the OPTIC trial (Dutch registry no. NL61668.041.17). The RASTRIC trial included patients with mCRC who were refractory to, or unable to receive, standard chemotherapeutic lines. In this trial a therapeutic combination was tested, thus patients had adequate bone marrow, kidney and liver function and capacity to ingest oral drugs. The OPTIC trial included patients with mCRC who receive standard of care treatment with chemotherapy and/or targeted agents, regardless of line of treatment and had given consent for inclusion in the Prospective Dutch Colorectal Cancer Cohort (PLCRC). Eligibility criteria for both studies were age of >18 years, metastases localized outside the bone, from which a biopsy can safely be obtained, metastases are measurable on CT imaging. Exclusion criteria included additional unrelated tumors influencing treatment decision making, potentially affecting size changes of metastases, or competing risk for survival. All patients in the chemonaive group were participants in the OPTIC trial. While these patients may have received adjuvant chemotherapy with a fluoropyrimidine with or without oxaliplatin, they were chemotherapy-naive for palliative treatments. Patients were selected based on availability of PDOs, complete clinical data, microsatellite stable status, and equal number of RAS and other driver mutations between groups based on sequencing data from standard clinical diagnostics. Detailed patient information is provided in Tables S1 and S2.

Both trials were approved by the Medical Research Ethics Committee (MREC) NedMec (OPTIC: #17–356; RASTRIC: #20–163), with the RASTRIC trial receiving additional evaluation from the Central Committee on Research Involving Human Subjects (CCMO). All patients provided written informed consent before participation in the respective trials.

#### Patient-derived organoids

Patient-derived organoids (PDOs) were established from 35 mCRC patients (18 pretreated, 17 chemonaive). PDO identity was confirmed using single-nucleotide polymorphism (SNP) array targeting 64 SNPs with TagMan OpenArray technology on the QuantStudio 12K Flex Real-Time PCR System (Utrecht Sequencing Facility). PDOs with a genetic distance <5 when comparing PDO DNA versus blood DNA were included in the study. PDOs were screened for mycoplasma.

### Method details

#### Organoid establishment and culture

PDO isolation was performed as described by Van de Wetering et al.^5^ PDOs were passaged using mechanical dissociation by pipetting and enzymatic dissociation with TrypLE Express (Gibco, Breda, the Netherlands, #12604021) for 5–10 min at 37°C, washed using Advanced DMEM supplemented with HEPES, Penicillin/Streptomycin and Glutamax (adDMEM+++) and re-plated by embedding in a solution of ice-cold BME (Bio techne, Dublin, Ireland, #3533-010-02) and plated as droplets on a pre-warmed 6-well plate. After solidification of the BME, organoid culturing medium was added to the plates. Culturing medium consisted of Advanced DMEM/F12 (Gibco, #12634028) supplemented with 10 mM HEPES (Santa Cruz Biotechnology, #SC-300789), 50 U/ml Penicillin/Streptomycin (Gibco, # 15070063), 2 mM GlutaMAX (Gibco, #35050-038), recombinant human R-spondin-3 conditioned medium, 100 ng/mL Noggin conditioned medium, 1x B27 (Gibco, #11530536), 1.25 mM N-acetylcysteine (Sigma-Aldrich, #A9165), 10 μM Y-27632 (MedChemExpress, #HY-10583), 500 nM A83-01 (MedChemExpress, #HY-10432), 0.05 nM Wnt surrogate (U-protein Express, #N001), 50 ng/mL human recombinant EGF (Sigma-Aldrich, #E9644), 100 ng/mL human recombinant IGF-I (BioLegend, #590908), and 50 ng/mL recombinant human FGF-basic (PeproTech, #100-18B). The organoid culture medium was refreshed two times per week.

#### Organoid drug screens

The timeline of the drug screen was as follows: On day −4 PDOs were passaged. On day 0, PDOs were harvested by incubating with 1 mg/mL Dispase (Life Technologies Europe B.V., Zuid-Holland, the Netherlands, #17105041) for 30 min at 37°C, washed twice using adDMEM+++ and filtered using a 100 μm mesh filter. PDOs were resuspended in organoid screening medium with 10% BME for a final 10 PDOs/μL concentration. The organoid screening medium was composed of the organoid culture medium without N-acetylcysteine and Rho-kinase inhibitor. Using an automated Multidrop Combi Reagent Dispenser, 40 μL of PDO suspension was dispensed in clear-bottomed, black-walled 384-well plates with ultra-low-attachment coating (Corning, Zuid-Holland, the Netherlands, #4588).

Drugs were tested in an 8-point logarithmic concentration range (Table S3). Technical triplicates were dispensed using a Tecan D300E dispenser. Negative controls contained either 1% Dimethyl Sulfoxide or matched concentrations of Tween as vehicle controls. All experimental conditions were normalized to contain consistent vehicle concentrations. The following standard-of-care CRC drugs were screened: 5-fluorouracil (5-FU, Accord, infusion concentrate, dissolved to a concentration of 100 mM with PBS to a final concentration containing 0.3% Tween), SN-38 (active metabolite of irinotecan, Selleck Chemicals, S4908), and oxaliplatin (Fresenius Kabi, infusion concentrate, dissolved to a concentration of 11.3 mM with PBS to a final concentration containing 0.3% Tween). The following drugs in phase 1/2 trials were screened: Alpelisib (Selleck Chemicals, S2814), Binimetinib (Selleck Chemicals, S7007), Lapatinib (Selleck Chemicals, S1028), Navitoclax (Selleck Chemicals, S1001), Prexasertib (Selleck Chemicals, S7178), vinorelbine (Selleck Chemicals, S4269). Combinations were screened by applying the following compounds together in ratios: SN-38 & prexasertib, alpelisib & lapatinib, alpelisib & binimetinib, binimetinib & lapatinib, navitoclax & vinorelbine and binimetinib & lapatinib & vinorelbine. On day 5, readouts were obtained by quantifying cell viability using CellTiter-Glo 3D (Promega, #G9681, 40μL/well) with a Tecan Spark plate reader with Spectramax. A baseline readout was measured on day 0 in a separate plate with PDOs without treatment to calculate growth rate inhibition (GR).^72^

Drug screens were performed in technical duplicates and mean values were calculated. The drug screen quality was analyzed by visualizing boxplots of positive and negative controls.^12^ Screens were repeated and excluded from the analysis if technical errors had occurred (e.g., dispensing error). The GR_AUC_ (area under the nonfitted ‘curve’ of the raw GR values) was calculated. Normalized values for GR_AUC_ were calculated using the maximum and minimum measured parameter per drug to compare different treatment types with different concentration ranges. Aggregated drug response curves were modeled by calculating the mean growth rate per concentration for all untreated and pretreated PDOs. Drug screen data are deposited in R2 at http://r2platform.com/strategic.^71^

#### RNA sequencing

Four-day-old PDOs were harvested and lysed in RLT-buffer (Qiagen) with 1% β-mercaptoethanol. RNA was isolated using QiaSymphony SP (Qiagen) and quality-checked (RNA Integrity Number >7) using an Agilent Bioanalyzer. Libraries were prepared with the Truseq RNA stranded polyA kit and sequenced on an Illumina NextSeq2000 (1x50 bp). Raw reads were preprocessed, aligned to GRCh38, and quantified. Batch correction was performed using limma (v3.60.0), with sample processing batch included as a categorical factor. For differential expression analysis we used DESeq2 (v1.44.0). Genes with counts below 10 in at least 4 samples were filtered out. For the gene expression correlation analysis with drug screen outcomes, we compared the five most resistant and sensitive PDOs for each drug (5-FU, SN-38, oxaliplatin). Genes with False Discovery Rate (FDR) < 0.05 and |log2FoldChange| > 1 were considered differentially expressed. Expression levels (limma batch-corrected) were correlated with drug sensitivity (GR_AUC_) using Pearson correlation, with FDR <0.1 considered significant. Gene Ontology analyses used g:Profiler with the TRANSFAC database. For visualizations ggplot2 was used. RNA sequencing data are deposited in R2 at http://r2platform.com/strategic.^71^

#### Whole genome sequencing

Four-day-old PDOs were harvested as described previously and frozen. Frozen PDO pellets and blood tubes were shipped to Hartwig Medical Foundation. Whole genome DNA sequencing was performed according to standard procedures described previously.^73^ DNA isolated from PDO pellets and blood was sequenced at depth of >30× using Illumina NovaSeq6000 WGS platform (2x 150bp). DNA from blood was used as a germline control. Sequencing data was analyzed with the Hartwig analytical processing pipeline (GitHub-hartwigmedical/pipeline5).^74^ Results from the PDO and germline sample are compared to filter out germline polymorphisms, which enables the reporting of somatic variants only. WGS data is deposited in R2 at http://r2platform.com/strategic.^71^ Code used for analysis of the WGS data is publicly available.^74^

#### Weighted gene co-expression network analysis (WGCNA)

WGCNA was performed using the WGCNA package (v1.71) in R. Gene expression data was filtered to retain the top 40% most variable genes to focus on informative expression patterns. A soft thresholding power was selected based on scale-free topology fit index (scale independence >0.8) and mean connectivity analysis. Network construction used the blockwiseModules function with the following parameters: signed network, minimum module size of 35 genes, deepSplit value of 2, and module merging threshold of 0.35 (correlation of 0.65). Module eigengenes (first principal component of each module) were calculated and correlated with clinical traits (pretreatment status, deep deletion status, and mutational signatures) and drug response metrics (normalized GR_AUC_ for 5-FU, oxaliplatin, and SN-38). Significant module-trait relationships were identified using Pearson correlation with *p* < 0.05. Functional enrichment analysis of significant modules was performed using g:Profiler with Gene Ontology, KEGG, and Hallmark gene sets to identify biological pathways associated with each module. For significant drug-associated modules, key hub genes were identified based on combined module membership and gene significance metrics.

#### Immunohistochemistry

Needle biopsies for histological analysis were obtained immediately following sample collection for PDO generation from the same patients during the same procedure. Tissue samples were formalin-fixed and paraffin-embedded (FFPE) before sectioning. PDOs were collected and washed with PBS before fixation in 4% paraformaldehyde for 30 min at room temperature. Following fixation, PDOs were washed twice with PBS and processed through graded ethanol dehydration (25%, 50%, 70% ethanol, 15 min each). PDOs were then embedded in 2% agar solution while warm and transferred to labeled tubes with PBS before submission for standard paraffin embedding and sectioning. Serial 4-μm sections were prepared from both tissue and PDO samples for histological and immunohistochemical analysis. To demonstrate morphological similarity between PDOs and corresponding tissue architecture, standard hematoxylin and eosin (H&E) staining was performed on both tissue sections and PDO sections according to routine histological protocols. To verify that PDO gene expression of L1CAM also translated to protein expression differences in patients, L1CAM immunohistochemical staining was performed. Sections underwent deparaffinization followed by antigen retrieval using CC1 buffer for 48 min. Primary antibody incubation was performed for 32 min at a 1:800 dilution. Immunodetection was completed using the OptiView DAB IHC Detection Kit, followed by hematoxylin counterstaining. All immunohistochemical procedures were performed on a Roche Ventana Ultra Benchmark automated staining platform.

#### Validation in clinical patient cohorts

To validate the clinical relevance of our gene expression findings, we analyzed publicly available patient data using the KM plotter tool (https://kmplot.com). We examined associations between gene expression and survival outcomes in stage 3 and 4 CRC patients who received chemotherapy. Survival analysis was performed using Cox proportional hazards regression with auto-selected cutoff values as described previously.^50^ Kaplan-Meier plots were generated to visualize associations between gene expression levels and overall survival.

### Quantification and statistical analysis

#### Statistical methods

Statistical analyses were performed in R (v4.0.3). Two-sided Pearson or Spearman correlation tests were used for continuous variables, with False Disovery Rate (FDR) correction using the Benjamini-Hochberg method. A two-sided T-test was used to compare groups. Dose-response modeling was conducted with nplr (v0.1-8), and statistical summaries and effect size calculations were performed using DescTools (v0.99.60). Differential gene expression analysis DESeq2 (v1.44.0) and batch correction was conducted using limma (v3.60.0). Co-expression network analysis was performed with WGCNA (v1.71). For differential expression analysis, significance was defined as FDR <0.05 and |log2FoldChange| > 1. For correlation analysis, significance was defined as FDR <0.1. For module–trait relationships in WGCNA, Pearson correlation coefficients were calculated and tested using a two-sided Student’s t test for correlation significance. Significance shown in heatmaps is based on nominal *p*-values (∗*p* < 0.05, ∗∗*p* < 0.01). Genomic data analysis was supported by GenomicRanges (v1.58.0), VariantAnnotation (v1.52.0), BSgenome (v1.74.0), and the BSgenome.Hsapiens.UCSC.hg38 genome reference (v1.4.5). Somatic mutation pattern analysis was performed using MutationalPatterns (v3.16.0). Data visualization was carried out using ggplot2 (v3.5.2), ggforce (v0.4.2), ggrepel (v0.9.6), ggalluvial (v0.12.5), and cowplot (v1.1.3) for multi-panel figure composition. Heatmaps were generated using pheatmap (v1.0.12) and ComplexHeatmap (v3.19).

### Additional resources

• RASTRIC trial: European registry no. 2019-004987-23
• OPTIC trial: Dutch registry no. NL61668.041.17

## Supplemental information

Document S1. Figures S1–S5 and Tables S1 and S2

Table S3. Detailed drug screen setup showing concentrations and combinations for all compounds

Table S4. Differentially expressed genes from all comparisons of the 5 most sensitive vs. the 5 most resistant PDOs for standard-of-care drugs

Table S5. Genes correlating with drug response (AUC) for standard-of-care drugs from all comparisons

Table S6. Genes in the black module, with their module membership value and correlation with the AUC of Oxaliplatin and SN38

Table S7. Genes in the brown module, with their module membership value and correlation with the AUC of Oxaliplatin and SN38

Table S8. Genes in the dark grey module, with their module membership value and correlation with AUC of Oxaliplatin and 5-FU

Table S9. Genes in the darkturquoise module, with their module membership value and correlation with AUC of Oxaliplatin and 5-FU

Table S10. Hallmark enrichment table for the modules: black, brown, darkgrey, and darkturquoise

Table S11. KEGG enrichment table for the modules: black, brown, darkgrey, and darkturquoise
