# Supplementary material for: Impact of treatment history on drug resistance of metastatic colorectal cancer organoids
Source: iScience. 2025 Oct 17;28(11):113801. doi: 10.1016/j.isci.2025.113801 (PMC12636388; doi:10.1016/j.isci.2025.113801)
Supplement: Document S1. Figures S1–S5 and Tables S1 and S2 [file mmc1.pdf]

iScience, Volume *28*

Supplemental information

Impact of treatment history on drug resistance of metastatic colorectal cancer organoids

Maarten A. Huismans, Lidwien P. Smabers, Sascha R. Brunner, Arne van Hoeck, Demi van de Kaa, Ingrid A. Franken, Emerens Wensink, Jan Koster, Richard Volckmann, Onno Kranenburg, Miriam Koopman, Hugo J.G. Snippert, and Jeanine M.L. Roodhart

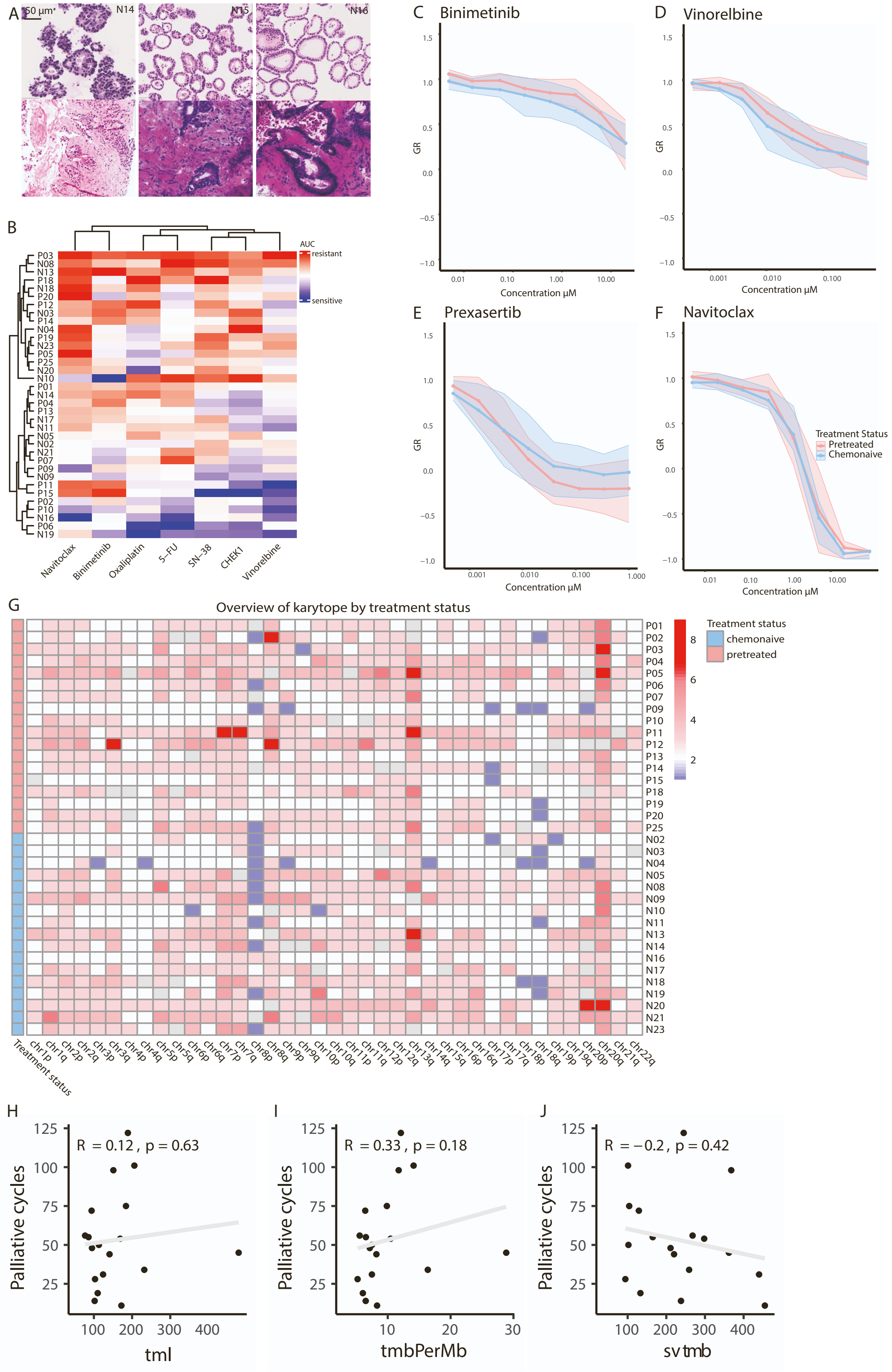

A
50 µm
N14
N15
N16
B
AUC
resistant
sensitive
P03
N08
N13
P18
N18
P20
P12
N03
P14
N04
P19
N23
P05
P25
N20
N10
P01
N14
P04
P13
N17
N11
N05
N02
N21
P07
P09
N09
P11
P15
P02
P10
N16
P06
N19
Navitoclax
Binimetinib
Oxaliplatin
5–FU
SN–38
CHEK1
Vinorelbine
C Binimetinib
D Vinorelbine
E Prexasertib
F Navitoclax
GR
Concentration µM
Treatment Status
Pretreated
Chemonaive
G
Overview of karytope by treatment status
Treatment status
chemonaive
pretreated
P01
P02
P03
P04
P05
P06
P07
P09
P10
P11
P12
P13
P14
P15
P18
P19
P20
P25
N02
N03
N04
N05
N08
N09
N10
N11
N13
N14
N16
N17
N18
N19
N20
N21
N23
chr1p
chr1q
chr2p
chr2q
chr3p
chr3q
chr4p
chr4q
chr5p
chr5q
chr6p
chr6q
chr7p
chr7q
chr8p
chr8q
chr9p
chr9q
chr10p
chr10q
chr11p
chr11q
chr12p
chr12q
chr13q
chr14q
chr15q
chr16p
chr16q
chr17p
chr17q
chr18p
chr18q
chr19p
chr19q
chr20p
chr20q
chr21q
chr22q
H
R = 0.12 , p = 0.63
Palliative cycles
tml
I
R = 0.33 , p = 0.18
tmbPerMb
J
R = −0.2 , p = 0.42
svtmb

Supplementary Figure 1. Histological and genomic characteristics for PDOs derived from chemonaive and pretreated mCRC patients.

A) Hematoxylin and eosin staining of three PDOs and corresponding patient biopsy tissues shows key histological features. Differences in tissue architecture and cellular organization show the similarities and variations between PDOs and metastasis. Scale bar=50 µm. B) Clustered heatmap of PDO drug response (normalized GR_{AUC}) to both experimental and standard-of-care drugs. C-F) Mean drug response curves for experimental therapies by treatment status. Y-axis shows growth rate (GR) metric, with 0 to 1 for partial growth inhibition, 0 for complete cytostasis, and 0 to −1 for cell death. Ribbons represent interquartile range. Experimental treatments shown are: C) Binimetinib, D) Vinorelbine, E) Prexasertib and F) Navitoclax. G) Heatmap of chromosomal copy number alterations (CNA). H-J) Correlation of number of chemotherapy cycles patients received with tumor mutational load (tml, H), tumor mutational burden per mega base (tmb per Mb, I), and structural variant tumor mutational burden per mega base (sv tmb, J) in PDOs. Two-sided Spearman correlation tests were used for continuous variables.

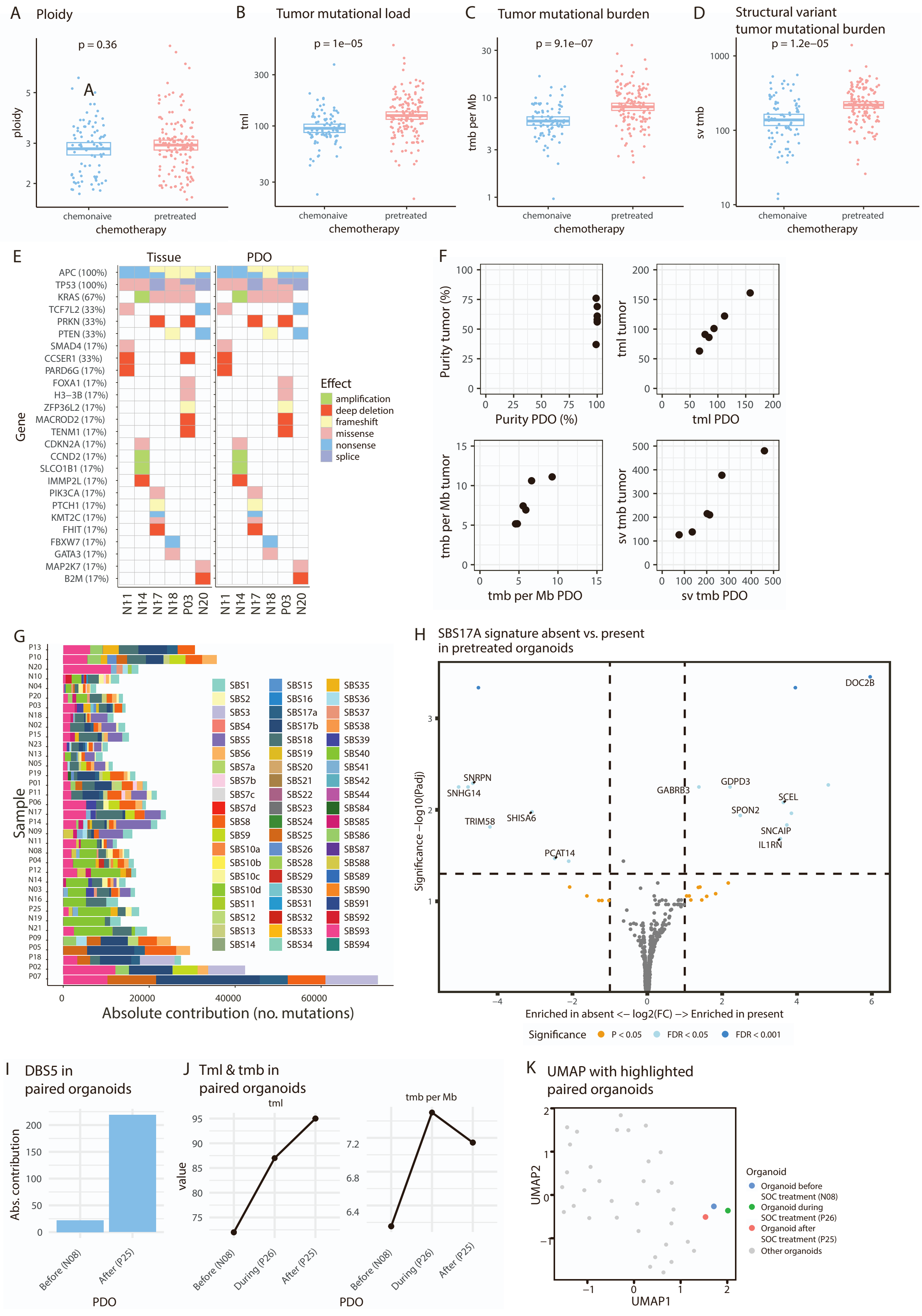

A Ploidy
p = 0.36
ploidy
chemonaive
pretreated
chemotherapy
B Tumor mutational load
p = 1e–05
tml
C Tumor mutational burden
p = 9.1e–07
tmb per Mb
D Structural variant tumor mutational burden
p = 1.2e–05
sv tmb
E
Tissue
PDO
Gene
APC (100%)
TP53 (100%)
KRAS (67%)
TCF7L2 (33%)
PRKN (33%)
PTEN (33%)
SMAD4 (17%)
CCSER1 (33%)
PARD6G (17%)
FOXA1 (17%)
H3–3B (17%)
ZFP36L2 (17%)
MACROD2 (17%)
TENM1 (17%)
CDKN2A (17%)
CCND2 (17%)
SLCO1B1 (17%)
IMMP2L (17%)
PIK3CA (17%)
PTCH1 (17%)
KMT2C (17%)
FHIT (17%)
FBXW7 (17%)
GATA3 (17%)
MAP2K7 (17%)
B2M (17%)
Effect
amplification
deep deletion
frameshift
missense
nonsense
splice
N11 N14 N17 N18 P03 N20
F
Purity tumor (%)
Purity PDO (%)
tml tumor
tml PDO
tmb per Mb tumor
tmb per Mb PDO
sv tmb tumor
sv tmb PDO
G
Sample
Absolute contribution (no. mutations)
H SBS17A signature absent vs. present in pretreated organoids
Significance –log10(Padj)
Enriched in absent <– log2(FC) –> Enriched in present
DOC2B
SNRPN
SNHG14
TRIM58
SHISA6
PCAT14
GABRB3
GDPD3
SCEL
SPON2
SNCAIP
IL1RN
Significance P < 0.05 FDR < 0.05 FDR < 0.001
I DBS5 in paired organoids
Abs. contribution
Before (N08)
After (P25)
PDO
J Tml & tmb in paired organoids
tml
tmb per Mb
value
Before (N08)
During (P26)
After (P25)
K UMAP with highlighted paired organoids
UMAP1
UMAP2
Organoid
Organoid before SOC treatment (N08)
Organoid during SOC treatment (P26)
Organoid after SOC treatment (P25)
Other organoids

Supplementary Figure 2. Genomic comparison of pretreated and chemonaive PDO groups and tumor tissue.

A-D) Comparison of genomic characteristics between pretreated and chemonaive patients: tumor ploidy (A), tumor mutational load (tml, B), tumor mutational burden per mega base (tmb per Mb, C), and structural variant tumor mutational burden per mega base (sv tmb, D). E) Comparison of most prevalent driver genes in patient tissue and PDOs ordered from most to least prevalent. F) Correlation of tumor purity, tumor mutational load (tml), tumor mutational burden per mega base (tmb per Mb), and structural variant tumor mutational burden per mega base (sv tmb) in patient tissue and PDOs. G) Absolute contribution of single-base substitution signatures to total tumor mutations by PDO sample. Samples are clustered based on similarity of their signature profile. H) Volcano plot of differential gene expression by presence of SBS17A signature in PDOs. Boxplots show the minimum, median, maximum, upper and lower quartiles, and individual data points. Y-axes of boxplots are on a logarithmic scale. I-J) DBS5 (I), tumor mutational load (tml) and tumor mutational burden per mega base (tmb per Mb, J) in PDOs derived from paired samples of the same patient before, during, and after systemic therapy. K) UMAP of the gene expression data of all PDOs, with highlighted paired PDOs.

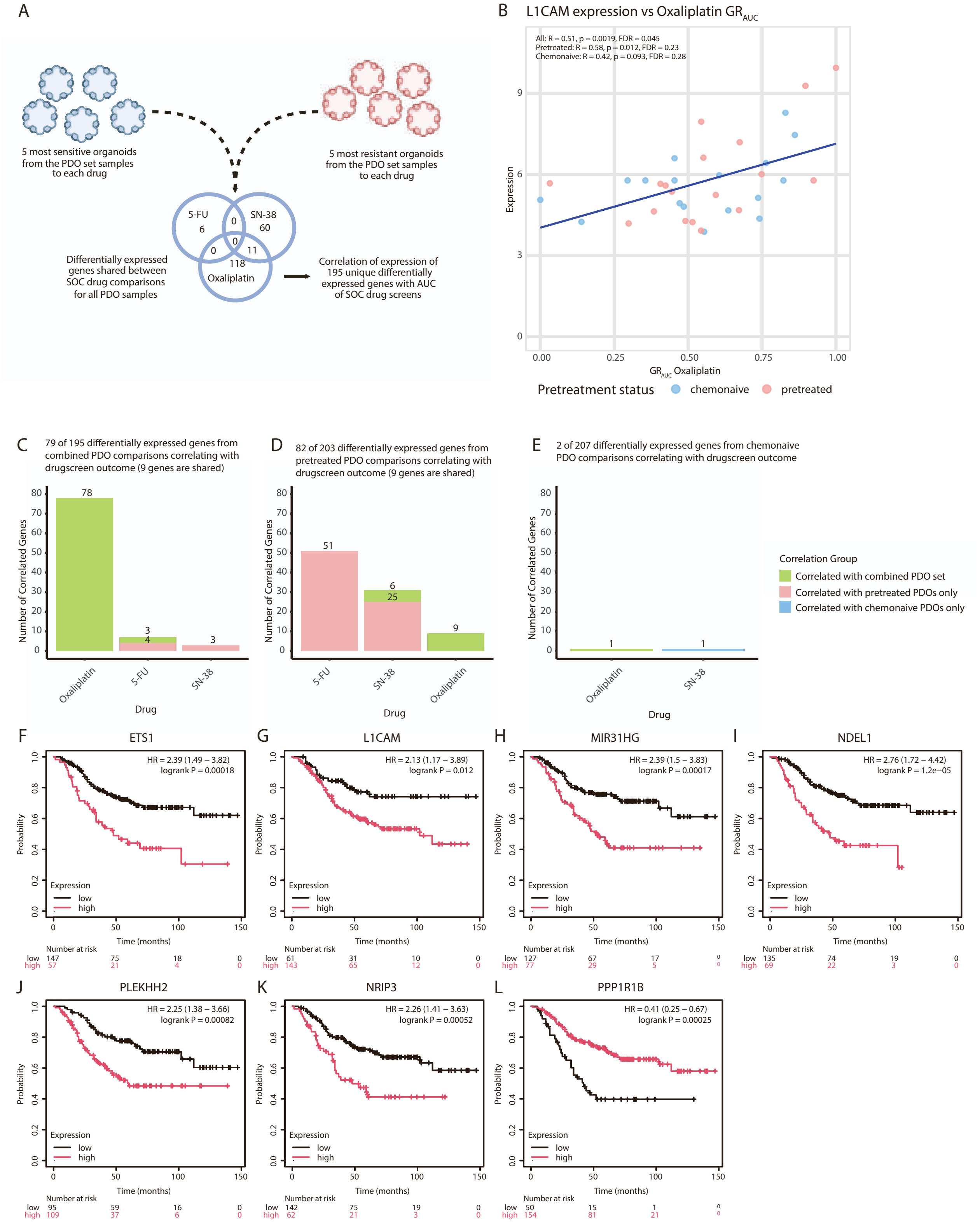
A
5 most sensitive organoids from the PDO set samples to each drug
5 most resistant organoids from the PDO set samples to each drug
5-FU 6
SN-38 60
Oxaliplatin 118
0
0
0
11
Differentially expressed genes shared between SOC drug comparisons for all PDO samples
Correlation of expression of 195 unique differentially expressed genes with AUC of SOC drug screens
B
L1CAM expression vs Oxaliplatin GR_AUC
All: R = 0.51, p = 0.0019, FDR = 0.045
Pretreated: R = 0.58, p = 0.012, FDR = 0.23
Chemonaive: R = 0.42, p = 0.093, FDR = 0.28
Expression
GR_AUC Oxaliplatin
Pretreatment status chemonaive pretreated
C
79 of 195 differentially expressed genes from combined PDO comparisons correlating with drugscreen outcome (9 genes are shared)
D
82 of 203 differentially expressed genes from pretreated PDO comparisons correlating with drugscreen outcome (9 genes are shared)
E
2 of 207 differentially expressed genes from chemonaive PDO comparisons correlating with drugscreen outcome
Number of Correlated Genes
Drug
Oxaliplatin
5-FU
SN-38
Correlation Group
Correlated with combined PDO set
Correlated with pretreated PDOs only
Correlated with chemonaive PDOs only
F ETS1 HR = 2.39 (1.49 – 3.82) logrank P = 0.00018
G L1CAM HR = 2.13 (1.17 – 3.89) logrank P = 0.012
H MIR31HG HR = 2.39 (1.5 – 3.83) logrank P = 0.00017
I NDEL1 HR = 2.76 (1.72 – 4.42) logrank P = 1.2e−05
J PLEKHH2 HR = 2.25 (1.38 – 3.66) logrank P = 0.00082
K NRIP3 HR = 2.26 (1.41 – 3.63) logrank P = 0.00052
L PPP1R1B HR = 0.41 (0.25 – 0.67) logrank P = 0.00025
Probability
Time (months)
Expression
low
high
Number at risk

Supplementary Figure 3. Differential gene expression analysis of sensitive versus resistant PDOs.

A) Venn diagram showing overlap of differentially expressed genes between 5-FU (6 genes), SN-38 (60 genes), and oxaliplatin (118 genes) comparisons. B) Correlation of L1CAM expression and PDO drug resistance (normalized GR_{AUC}) to oxaliplatin. Each point represents an individual PDO, color-coded by pretreatment status (chemonaive in blue, n=17, pretreated in red, n=18). Two-sided Pearson correlation tests were used. C-E) Bar plot showing numbers of differentially expressed genes correlating with drug sensitivity of each drug: 78 genes with oxaliplatin, 7 with 5-FU, 3 with SN-38. Color indicates if the genes were correlated with drug sensitivity in the full PDO set (green), or only in the pretreated (pink) or chemonaive (blue) subset. Differentially expressed genes are shown between the 5 most sensitive versus the 5 most resistant PDOs of the full PDO set (C), the pretreated subset (D), and the chemonaive subset (E). F-L) Kaplan-Meier survival curves for overall survival in stage 3 and 4 CRC patients who received chemotherapy, stratified by high (red) or low (black) expression of identified genes: ETS1 (F), L1CAM (G), MIR31HG (H), NDEL1 (I), PLEKHB2 (J), NR1P3 (K), and PPP1R1B (L). Hazard ratios (HR), 95% confidence intervals, and logrank p-values are shown for each gene.

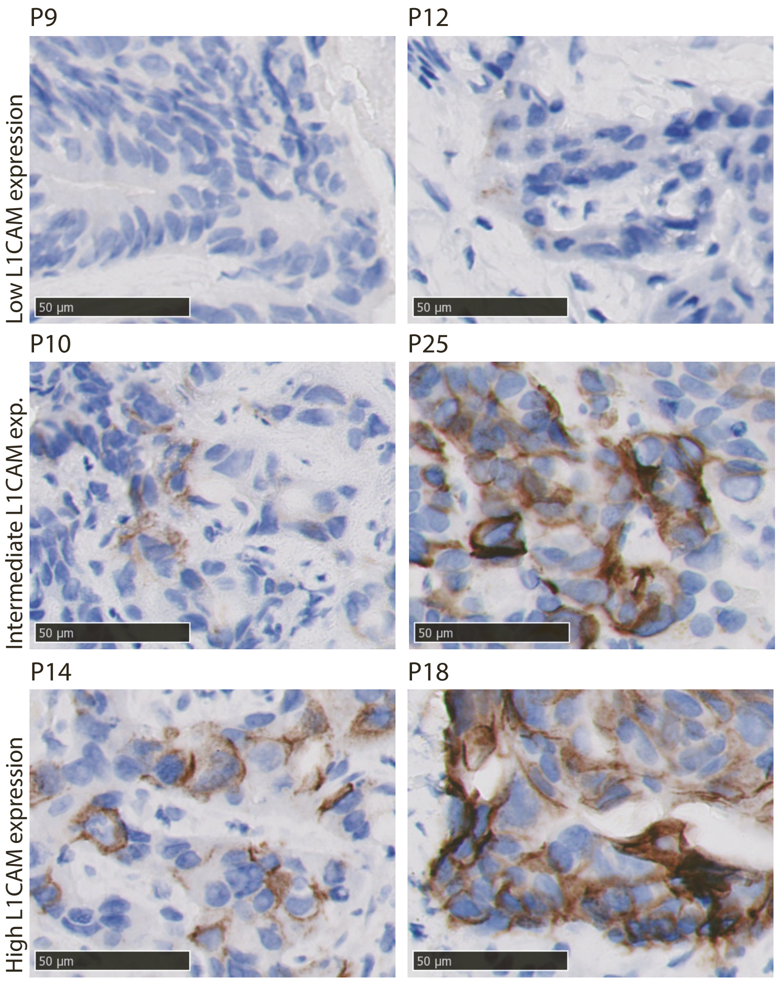

P9
P12
P10
P25
P14
P18
Low L1CAM expression
Intermediate L1CAM exp.
High L1CAM expression
50 µm

Supplementary Figure 4. Immunohistochemical staining of L1CAM in different pretreated PDOs.

Representative DAB immunohistochemical staining of L1CAM in patient biopsy tissues. Upper panels show tissue from patients whose corresponding PDOs had low L1CAM normalized expression (P9, P12: range 5-11). Middle panels show intermediate expression (P10, P25: range 36-78). Lower panels show high L1CAM expression (P14, P18: range 259-1575). Scale bar=50 µm.

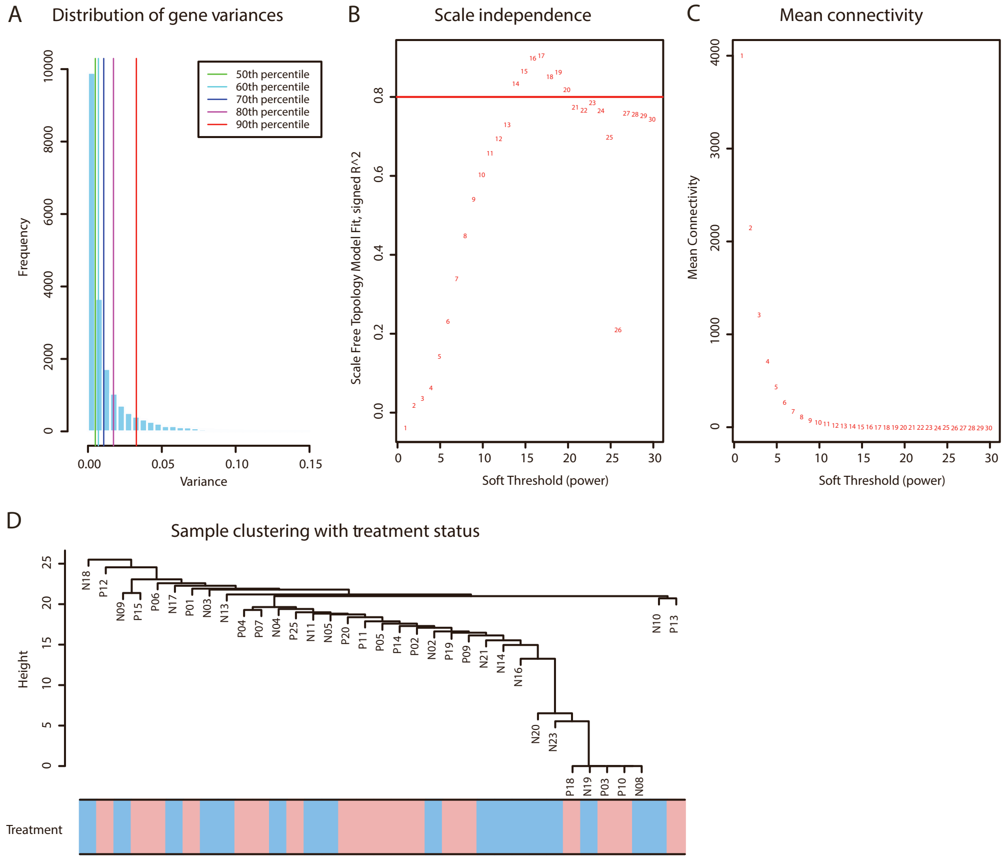

A
Distribution of gene variances
Frequency
Variance
50th percentile
60th percentile
70th percentile
80th percentile
90th percentile
B
Scale independence
Scale Free Topology Model Fit, signed R^2
Soft Threshold (power)
C
Mean connectivity
Mean Connectivity
Soft Threshold (power)
D
Sample clustering with treatment status
Height
Treatment

Supplementary Figure 5. WGCNA network construction and module characterization.

A) Histogram showing gene variance distribution with percentile lines indicating filtering thresholds. B-C) Scale independence and mean connectivity plots for determining optimal soft thresholding power. D) Hierarchical clustering dendrogram of PDO samples based on gene expression, with pretreatment status indicated by color.

Table S1. Extended clinical data of patients from the chemonaive PDO cohort

ID	Cohort	Age	Sex	Primary Tumor location	ECOG PS	RAS mutational status	Site of biopsy	Time to metastases	OS	Adjuvant treatment
N02	OPTIC	71	Female	Right-sided	1	WT	Liver	Metachronous	2 months	Adjuvant capecitabine and oxaliplatin
N03	OPTIC	68	Male	Right-sided	0	KRAS p.G12D	Liver	Synchronous	14 months	No adjuvant treatment
N04	OPTIC	72	Female	Left-sided	1	WT	Liver	Metachronous	47 months	Adjuvant capecitabine and oxaliplatin
N05	OPTIC	54	Male	Right-sided	1	KRAS p.G12V	Liver	Synchronous	0 months	No adjuvant treatment
**N08*	OPTIC	53	Male	Rectum	0	KRAS p.G12C	Liver	Synchronous	21 months	No adjuvant treatment
N09	OPTIC	69	Male	Rectum	0	KRAS p.G12V	Liver	Synchronous	27 months	No adjuvant treatment
N10	OPTIC	86	Female	Left-sided	1	KRAS p.G12D	Liver	Metachronous	25 months	No adjuvant treatment
N11	OPTIC	37	Female	Rectum	3	WT	Liver	Synchronous	17 months	No adjuvant treatment
N13	OPTIC	74	Male	Right-sided	Unknown	KRAS	Liver	Synchronous	1 month	No adjuvant treatment
N14	OPTIC	69	Male	Rectum	1	WT gene amplificiation	Liver	Metachronous	41 months	Received an unkown adjuvant treatment
N16	OPTIC	79	Female	Left-sided	1	KRAS p.G12D	Liver	Synchronous	4 months	No adjuvant treatment
N17	OPTIC	44	Male	Right-sided	1	KRAS p.G13D	Liver	Synchronous	36 months	No adjuvant treatment
N18	OPTIC	46	Female	Rectum	Unknown	KRAS p.K117N	Liver	Synchronous	22 months	No adjuvant treatment
N19	OPTIC	80	Male	Left-sided	1	KRAS p.G12C	Liver	Synchronous	13 months	No adjuvant treatment
N20	OPTIC	70	Male	Left-sided	1	WT	Liver	Synchronous	0 months	No adjuvant treatment
N21	OPTIC	72	Female	Right-sided	2	NRAS p.Q61H	Liver	Synchronous	2 months	No adjuvant treatment
N23	OPTIC	49	Male	Rectum	0	KRAS p.G12V	Liver	Synchronous	17 months	No adjuvant treatment

Table S2. Extended clinical data of patients from the pretreated PDO cohort

ID	Cohort	Age	Sex	Primary Tumor location	ECOG PS	RAS mutational status	Site of biopsy	Time to metastases	OS	Adjuvant treatment	Palliative treatment	Palliative treatment (months)
P01	OPTIC	66	Male	Left-sided	0	KRAS p.G12A	Liver	Metachronous	42	Unkown	5-FU (25 cycles, PD), irinotecan (6 cycles, PR), did not receive oxaliplatin prior to biopsy, but was progressive directly after	37
P02	OPTIC	71	Male	Left-sided	0	WT	Liver	Metachronous	52	Unkown	5-FU (13 cycles, PD), irinotecan (14 cycles, PD), oxaliplatin (7 cycles, SD or PR)	50
P03	OPTIC	40	Female	Rectum	0	KRAS p.G12D	Liver	Synchronous	27	Unkown	5-FU (30 cycles, PD), irinotecan (17 cycles, PD), oxaliplatin (9 cycles, PD)	22
P04	OPTIC	68	Female	Left-sided	0	WT	Liver	Synchronous	8	Unkown	5-FU (7 cycles, PD), irinotecan (4 cycles, PD) oxaliplatin (3 cycles, PD)	6
P05	OPTIC	69	Male	Left-sided	0	WT	Liver	Synchronous	82	Unkown	5-FU (56 cycles, PD), irinotecan (30 cycles, PD), oxaliplatin (12 cycles, SD)	69
P06	OPTIC	74	Male	Left-sided	0	WT	Liver	Synchronous	43	Unkown	5-FU (26 cycles, PD), irinotecan (18 cycles, PR), oxaliplatin (6 cycles, PR)	27
P07	OPTIC	83	Male	Left-sided	0	KRAS p.K117N	Liver	Metachronous	51	Adjuvant capecitabine and oxaliplatin	5-FU (31 cycles, PD), did not receive irinotecan prior to biopsy, but was progressive directly after, oxaliplatin (10 cycles, PD)	39
P09	RASTRIC	51	Male	Rectum	1	KRAS p.Q61H	Liver	Metachronous	61	Neoadjuvant chemoradiation (capecitabine)	5-FU (46 cycles, PD), irinotecan (14 cycles, PD), oxaliplatin (15 cycles, PD)	52
P10	RASTRIC	58	Male	Left-sided	0	KRAS p.G12V	Liver	Metachronous	67	No adjuvant treatment	5-FU (69 cycles, PD), irinotecan (13 cycles, PD), oxaliplatin (19 cycles, SD)	53
P11	RASTRIC	68	Male	Left-sided	1	KRAS p.G12A	Skin	Metachronous	49	No adjuvant treatment	5-FU (24 cycles, PD), irinotecan (9 cycles, PD), oxaliplatin (11 cycles, SD)	46
P12	RASTRIC	48	Female	Rectum	0	KRAS p.G12C	Lymph node	Metachronous	62	Adjuvant capecitabine and oxaliplatin	5-FU (28 cycles, PD), irinotecan (12 cycles, PD), oxaliplatin (15 cycles, PD)	59
P13	RASTRIC	41	Female	Left-sided	0	WT (amp)	Lung	Metachronous	64	No adjuvant treatment	5-FU (51 cycles, PD), irinotecan (30 cycles, PD), oxaliplatin (41 cycles, SD)	50
P14	RASTRIC	55	Female	Left-sided	1	KRAS p.A146P	Liver	Synchronous	9	No adjuvant treatment	5-FU (4 cycles, PD), irinotecan (3 cycles, PD) oxaliplatin (4 cycles, PD)	5
P15	RASTRIC	54	Male	Left-sided	1	KRAS p.G12C	Liver	Metachronous	21	No adjuvant treatment	5-FU (8 cycles, PD), irinotecan (5 cycles, PD) oxaliplatin (6 cycles, SD)	16

P18	RASTRIC	62	Male	Rectum	1	KRAS p.G12V	Lung	Metachronous	34	No adjuvant treatment	5-FU (38 cycles, PD), irinotecan (8 cycles, PD), oxaliplatin (8 cycles, PD)	30
P19	RASTRIC	46	Male	Left-sided	1	KRAS p.G12D	Liver	Synchronous	26	No adjuvant treatment	5-FU (24 cycles, PD), irinotecan (24 cycles, PR), oxaliplatin (24 cycles, PR)	22
P20	RASTRIC	59	Male	Rectum	1	KRAS p.G12V	Liver	Synchronous	18	No adjuvant treatment	5-FU (12 cycles, PD), irinotecan (12 cycles, PD), oxaliplatin (4 cycles, SD)	11
**P25*	RASTRIC	55	Male	Rectum	1	KRAS p.G12C	Liver	Synchronous	21	No adjuvant treatment	5-FU (21 cycles, PD), irinotecan (15 cycles, PD), oxaliplatin (12 cycles, PD)	18
**P26*	RASTRIC	55	Male	Rectum	1	KRAS p.G12C	Liver	Synchronous	21	No adjuvant treatment	5-FU (16 cycles, PD), irinotecan (15 cycles, PD), oxaliplatin (7 cycles, PD)	16

*P25, P26, and N08 are PDOs established from different biopsies from the same patient at different stages of this patient's treatment: before (N08), after two lines of palliative chemotherapy, but before a third line of 5-FU and oxaliplatin (P26), and after all standard of care therapy lines (P25).
